# Freehand Needle Hybrid Brachytherapy in Complex Gynecological Cancers

**DOI:** 10.7759/cureus.116998

**Published:** 2026-09-27

**Authors:** Sadia Sadiq, Rafia Usman, Asia Razzaq, Rab Nawaz Maken

**Affiliations:** 1 Radiation Oncology, Institute of Nuclear Medicine and Oncology (INMOL) Atomic Energy Cancer Hospital, Lahore, PAK; 2 Oncology, Institute of Nuclear Medicine and Oncology (INMOL) Atomic Energy Cancer Hospital, Lahore, PAK; 3 Medical Physics, Institute of Nuclear Medicine and Oncology (INMOL) Atomic Energy Cancer Hospital, Lahore, PAK

**Keywords:** freehand needles, gynecological cancers, hybrid brachytherapy, interstitial brachytherapy, resource limited setting

## Abstract

Hybrid intracavitary-interstitial brachytherapy improves local control in complex gynecologic malignancies. However, access to advanced applicators remains limited in resource-constrained settings. We present a case series of three patients with challenging anatomical and disease characteristics in whom personalized hybrid brachytherapy was performed using freehand interstitial needle placement. All patients received standard chemoradiation followed by CT-guided brachytherapy. Due to inadequate coverage with conventional applicators, individualized freehand needle placement was performed and stabilized using locally available materials. Adequate dosimetric coverage of the high-risk clinical target volume was achieved in all cases while respecting organ-at-risk constraints. Two patients demonstrated a complete response on follow-up imaging, while one showed excellent clinical response. This report highlights a practical, cost-effective alternative when advanced templates are unavailable and underscores the importance of innovation in resource-limited environments.

## Introduction

Cervical and endometrial cancers represent major public health challenges in low- and middle-income countries (LMICs), where access to screening, timely diagnosis, and advanced treatment facilities remains limited. According to GLOBOCAN 2022, cervical cancer is the eighth most commonly diagnosed cancer worldwide and remains disproportionately prevalent in low-income countries, where it is among the leading causes of cancer-related mortality in women [1]. In Pakistan, an estimated 4,762 new cervical cancer cases and 3,069 deaths occurred in 2022, reflecting persistent gaps in human papillomavirus (HPV) vaccination, awareness, screening, and timely access to care, with many patients consequently presenting with advanced-stage disease [1,2].

Brachytherapy, in which radioactive sources are placed close to or within the tumor to deliver a highly localized radiation dose, remains an integral component of curative treatment for locally advanced cervical cancer and selected cases of recurrent endometrial cancer [3-5]. In intracavitary brachytherapy, an applicator is positioned within the uterus and vagina, allowing the radiation source to deliver dose from within the body cavities. Conventional intracavitary brachytherapy using tandem-and-ovoid or ring applicators provides adequate target coverage in many patients. However, tumors with bulky, asymmetric, lateral, deep vaginal, or parametrial extension, as well as altered anatomy following surgery, may extend beyond the dose distribution achievable with a standard intracavitary applicator [6,7]. When the applicator geometry does not conform to the shape or extent of the tumor, portions of the target may receive an inadequate radiation dose, potentially compromising local tumor control.

Interstitial brachytherapy addresses this limitation by placing needles directly into or adjacent to the tumor, allowing radioactive sources to be positioned within the tissues beyond the reach of the intracavitary applicator. Hybrid brachytherapy combines intracavitary and interstitial components, thereby allowing the advantages of both approaches to be used simultaneously. The addition of interstitial needles can improve the conformity of the high-dose region to irregularly shaped or laterally extending tumors and facilitate adequate target coverage while respecting the dose constraints of surrounding organs at risk. Hybrid techniques have therefore demonstrated improved dosimetric coverage and favorable local control in appropriately selected patients without excessive toxicity [8,9].

Commercial hybrid applicators and dedicated templates have facilitated the incorporation of interstitial needles into gynecologic brachytherapy. However, their cost, availability, and requirement for specialized equipment and training may limit their use in resource-constrained centers. Three-dimensional (3D)-printed or customized templates can provide a reproducible means of guiding needle placement, while freehand interstitial needle placement represents a more pragmatic approach when dedicated templates or commercial hybrid applicators are unavailable [10,11]. Despite the potential utility of such approaches, there is limited published evidence describing practical freehand needle techniques that can be implemented using locally available materials in resource-limited settings.

In this case series, we describe three complex gynecologic cancer cases in which individualized hybrid brachytherapy was performed using freehand interstitial needle placement stabilized with locally available materials. At our institution, computer tomography (CT)-guided brachytherapy is performed under aseptic measures and deep sedation as a day procedure. Magnetic resonance imaging (MRI) guidance is not utilized routinely due to resource, time, and financial constraints. When available templates were insufficient to cover the extensive disease in these cases, we planned to use freehand needles and counselled the patients regarding the complexity of the procedure, unavailability of advanced templates, and treatment outcomes along with expected side effects. We placed individual needles under visual or finger guidance to make personalized templates and devised the method of using Styrofoam and medical tape to secure the needles, ensuring reproducible positioning and optimal dose distribution. The specific geometry was recorded along with the length of needles from the perineum to ensure precise treatment delivery. High-risk clinical target volume (HR-CTV) and organs at risk (OARs) were delineated, and patients underwent CT-based treatment planning. We prioritized achieving a high dose to the HR-CTV while limiting doses to critical OARs such as the bladder, rectum, and sigmoid colon. Cumulative EQD2 (equivalent doses in 2 Gy per fraction) doses were calculated using a Microsoft Excel-based (Microsoft Corporation, Redmond, WA, USA) spreadsheet, applying an α/β ratio of 10 Gy for the HR-CTV and 3 Gy for the OARs.

These cases illustrate a pragmatic approach to achieving adequate target coverage in challenging anatomies when conventional applicator geometry is insufficient and dedicated hybrid applicators or templates are unavailable.

## Case presentation

Case 1

A 68-year-old female patient diagnosed with recurrent endometrial cancer with deep right vaginal wall involvement, International Federation of Gynecology and Obstetrics (FIGO) Stage IIIB [12]. The patient underwent a simple hysterectomy in September 2021 for high-grade endometrioid adenocarcinoma involving more than half of the myometrium, with positive lymphovascular space invasion. She did not receive adjuvant treatment, and two years later, in August 2023, she presented to us with recurrent symptoms. Examination revealed a normal vaginal cuff with a lesion involving the middle two-thirds of the right vaginal wall with restricted mobility. MRI demonstrated a heterogeneously enhancing mass along the right vaginal wall extending into pelvic wall muscles. CT staging ruled out distant metastases and lymphadenopathy. Biopsy confirmed high-grade adenocarcinoma (PAX8+, ER+, P53+). After four cycles of carboplatin/paclitaxel chemotherapy starting in November 2023**,** the patient received concomitant chemoradiation with 45 Gy in 25 fractions and weekly Cisplatin 40 mg/m^2^. At the time of the first brachytherapy, there was residual disease present, and adequate tumor coverage was not possible with the vaginal cylinder alone, as a simple cylinder only covers 5 mm of mucosal depth. Commercially available perineal templates are useful for deep vaginal disease coverage, but none was available at our institution. So, we palpated the disease and used tactile guidance for placing three individual needles in the right vaginal wall lesion and used Styrofoam to stabilize the geometry (Figure 1). We achieved good tumor coverage and repeated the same for the remaining fractions. The patient received four fractions of 7 Gy each. Cumulative EQD2 D90 of HR-CTV was 94.2 Gy, and D2cc (dose to the most exposed 2 cm^3^ of the organ at risk) of the bladder and rectum were 66 Gy and 60.9 Gy, respectively. After three months, clinical examination showed mild fibrotic changes in the right vaginal wall, and the MRI pelvis showed a complete response. During follow-up, she developed symptoms of vaginitis and was treated conservatively. She remained disease-free until October 2025, when she developed pulmonary metastasis and is currently receiving palliative systemic therapy.

**Figure 1:**
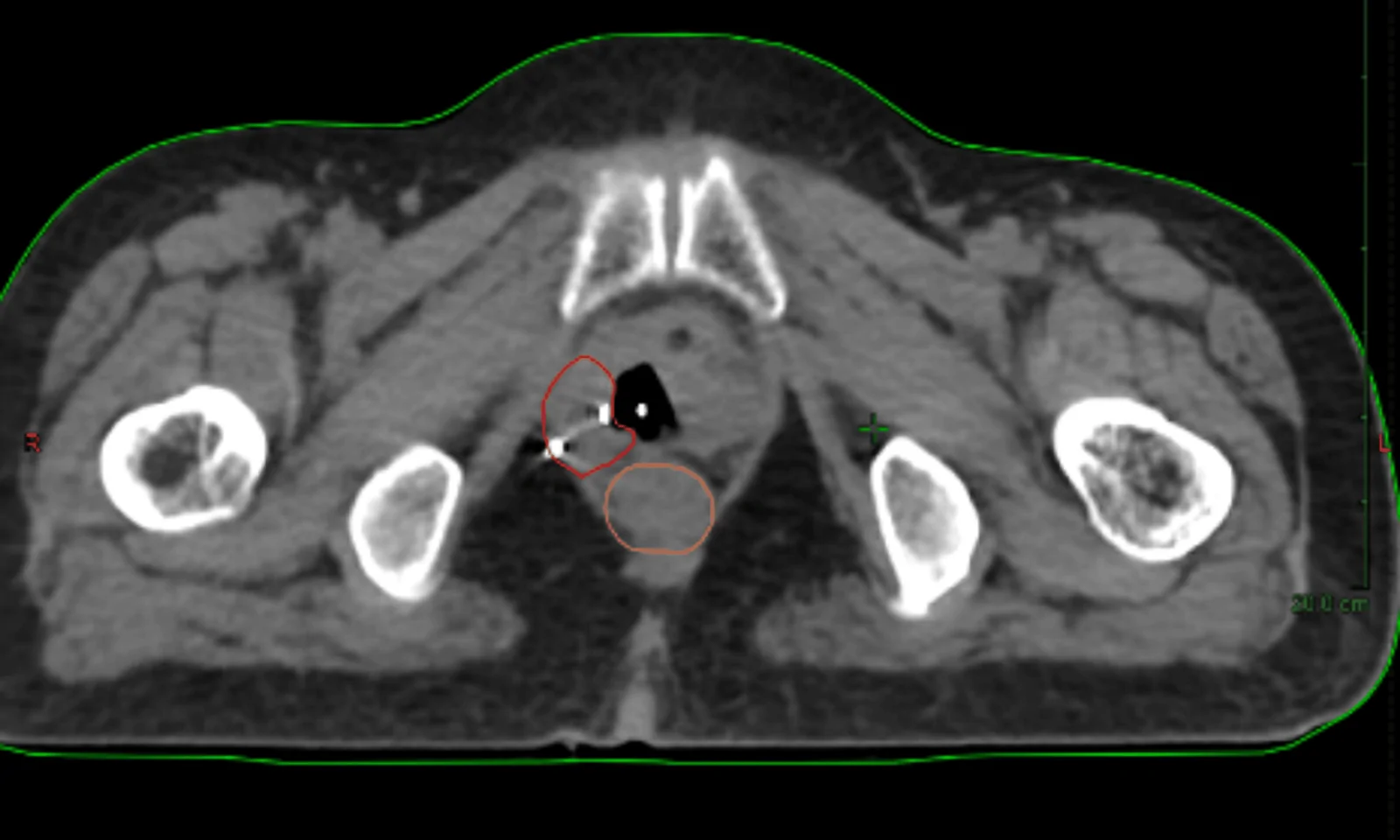
Axial CT image from Case 1 showing three needles in right vaginal wall.

Case 2

A 45-year-old female patient underwent subtotal hysterectomy for locally advanced cervical cancer in a private medical center in February 2024. No preoperative cross-sectional imaging was performed, and the histopathology could not be done as the sample was lost. After three months, the patient presented to us with recurrent symptoms. Examination revealed a large cauliflower-like mass on the vaginal stump involving vaginal walls up to the lower third of the vagina. The lesion was extending to the vulvar area, involving the clitoris and adjacent parts of the labia minora. MRI of the pelvis demonstrated a heterogeneous mass involving the vaginal stump and bilateral vulva. Fat planes around the bladder and rectum were preserved. There were no enlarged lymph nodes, and it was FIGO Stage IIIA disease. The biopsy confirmed moderately differentiated squamous cell carcinoma (p63+ve, p40+ve, p16+ve). The patient was planned for concomitant chemoradiation, and she received pelvic radiation of 45 Gy in 25 fractions with weekly cisplatin in August 2024. At the time of the first brachytherapy, there was persistent disease involving the vagina and vulva with minimal treatment response. This was a unique case as we did not have experience with vulval brachytherapy at our institution due to specific template and anesthesia unavailability. We discussed this among colleagues, and in order to cover this extensive disease, we used a vaginal cylinder with interstitial needles to cover vaginal disease and placed three direct clitoral needles stabilized with Styrofoam (Figure 2). She was planned for four insertions of 7 Gy each. The EQD2 of the D90 HR-CTV was 88.2 Gy, and the D2cc of the bladder, rectum, and sigmoid colon was 83 Gy, 74 Gy, and 73.5 Gy, respectively. She had a good clinical response with a substantial decrease in the size of the vaginal and clitoral lesion at the time of the last brachytherapy fraction and at three-month follow-up. A radiological assessment could not be completed as she did not undergo a response evaluation MRI due to financial constraints. Her last documented follow-up is from November 2025, when she had purulent vaginal discharge and was prescribed antibiotics.

**Figure 2:**
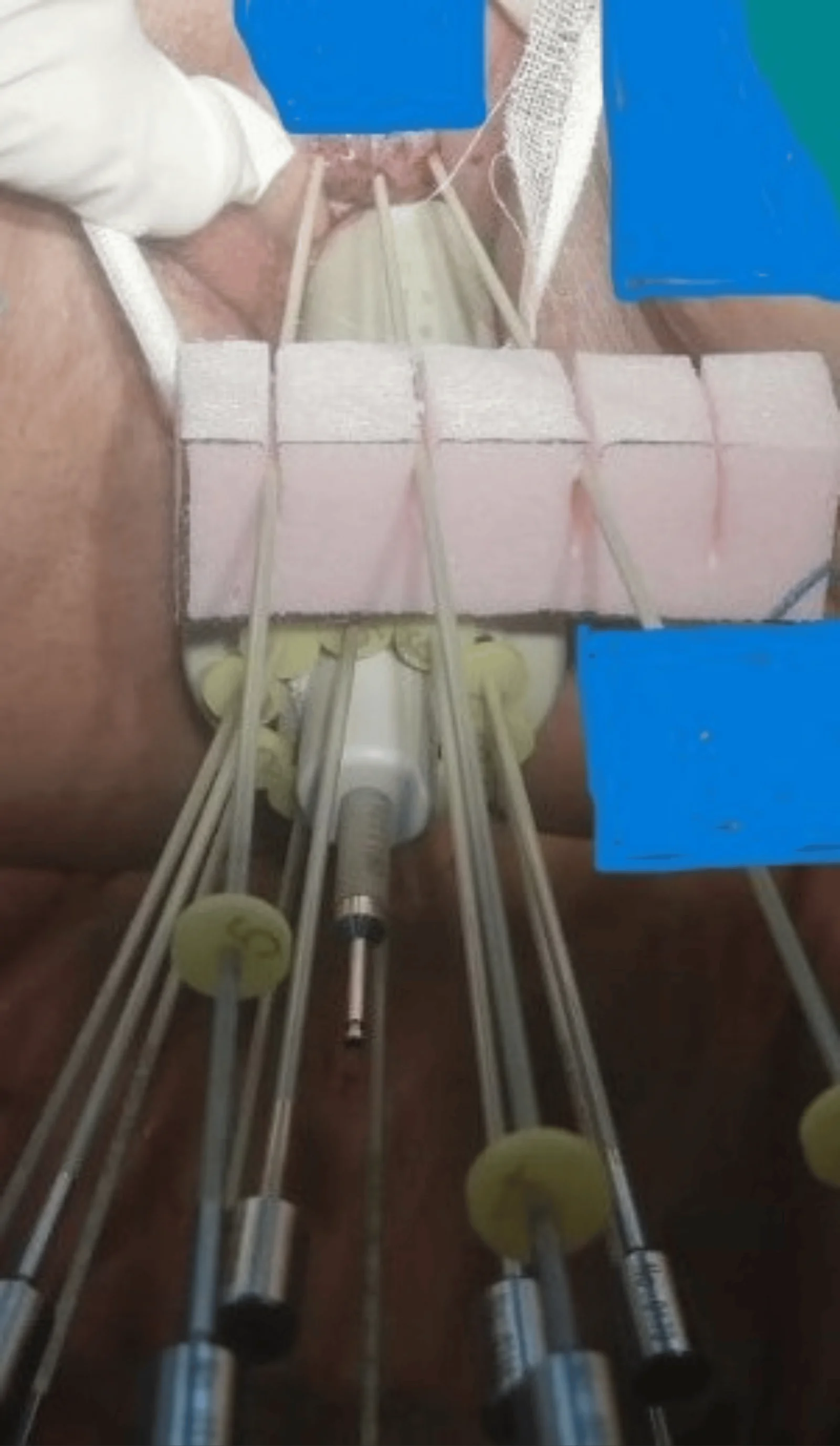
Hybrid interstitial implant in Case 2 showing a vaginal cylinder with interstitial needles and additional freehand needles directed toward clitoral disease extension to achieve adequate target coverage.

Case 3

A 65-year-old female patient with locally advanced cervical cancer with unilateral extensive parametrial involvement. She presented in October 2023 with a six-month history of heavy post coital bleeding and anemia. On bimanual pelvic examination, she had an enlarged uterus with restricted mobility and a large nodular cervical mass involving the middle third of the vagina. Rectal examination showed left parametrial involvement extending to the pelvic sidewall. Pelvic MRI confirmed similar findings with multiple enlarged left pelvic lymph nodes, FIGO Stage IIIC. Biopsy was positive for poorly differentiated squamous cell carcinoma.

The patient received external beam pelvic radiation 45 Gy in 25 fractions with a simultaneous integrated 55 Gy boost to enlarged lymph nodes in November 2023. Examination before brachytherapy showed a limited treatment response. Available templates were insufficient to cover the deep lateral disease extent, so we used a hybrid approach consisting of a conventional Fletcher-Suit applicator with placement of left-sided freehand needles stabilized with Styrofoam (Figure 3).

**Figure 3:**
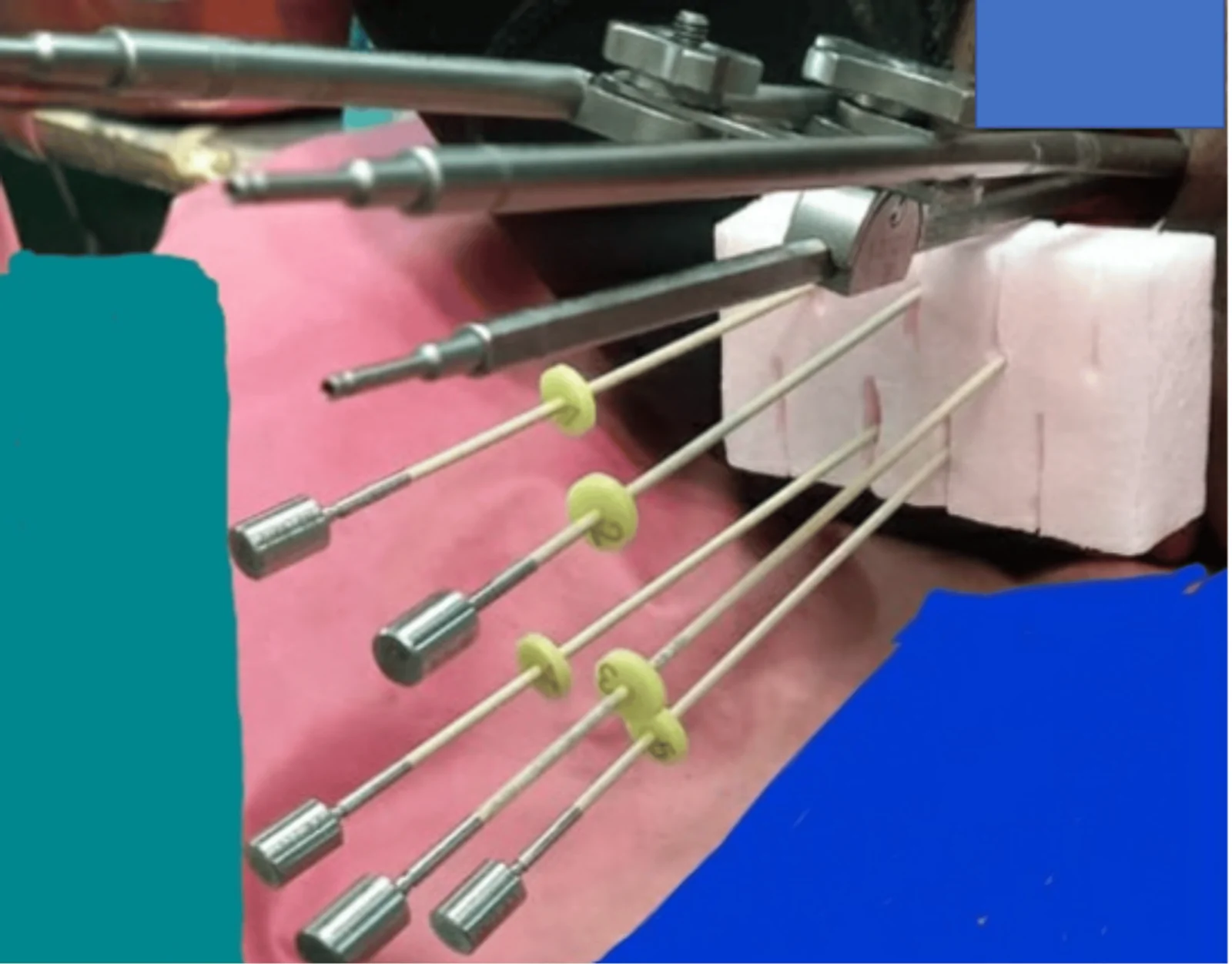
Hybrid brachytherapy applicator arrangement in Case 3. Multiple freehand interstitial needles were inserted to cover extensive left parametrial disease and stabilized using Styrofoam blocks to maintain needle geometry during treatment planning and delivery.

After four fractions of brachytherapy, EQD2 turned out to be 81.2 Gy for HR-CTV, with D90 and D2cc of 82 Gy, 75 Gy, and 74.5 Gy for bladder, rectum, and sigmoid colon, respectively. She had a good response, with MRI after three months showing a 2.5 cm residual cervical mass. A repeat MRI at six months showed complete response with no residual disease (Figure 4). The patient developed grade 2 proctitis, according to Radiation Therapy Oncology Group (RTOG) criteria [13], in March 2025 and was managed with dietary modifications, steroid enemas, and two procedures of argon plasma coagulation. She is alive and disease-free with no significant bowel or urinary complaints, according to the latest patient encounter documented in December 2025.

**Figure 4:**
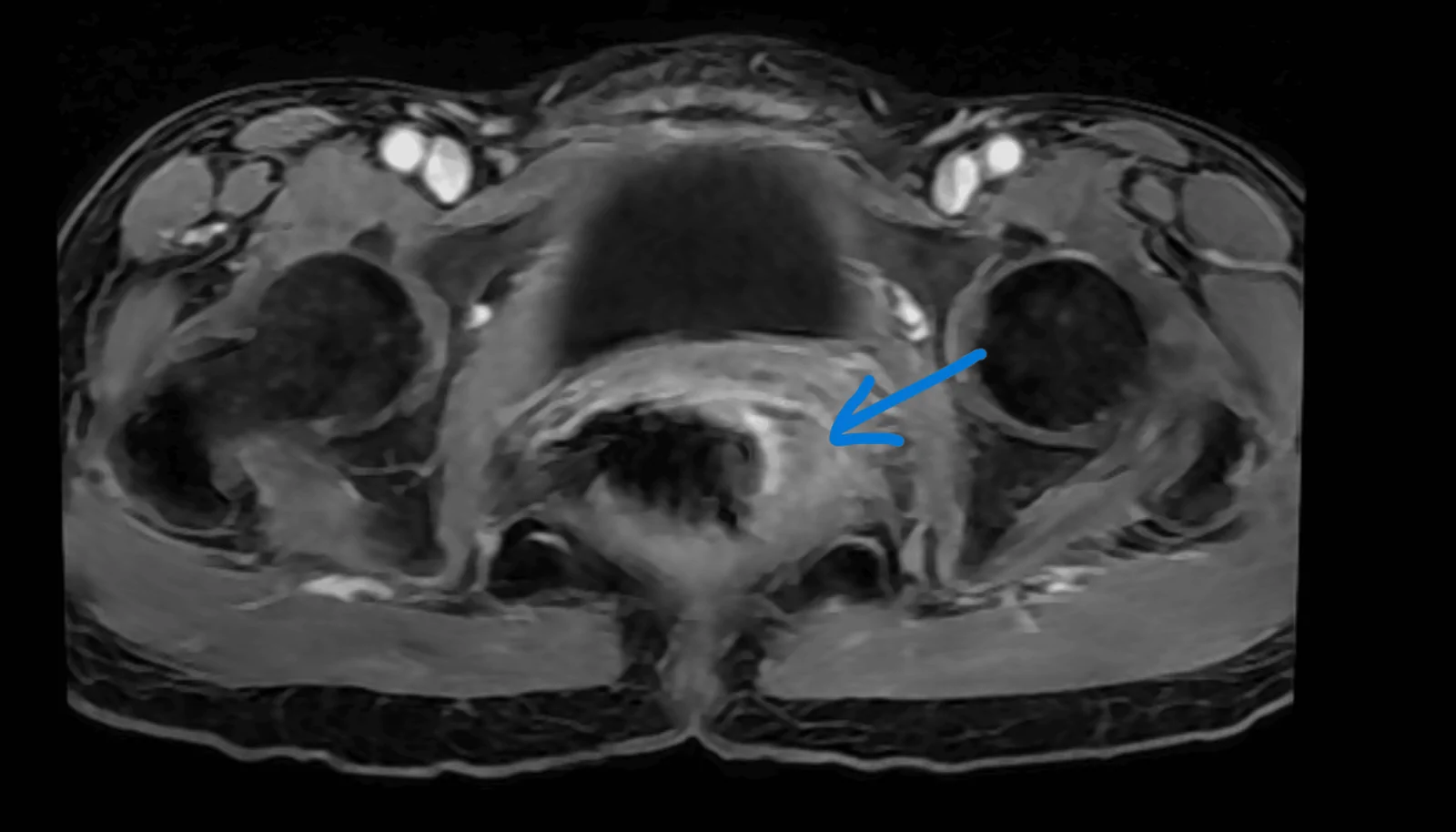
Axial image of MRI of Case 3, with the blue arrow indicating previously involved left parametrial region, with no residual measurable tumor.

## Discussion

For locally advanced cervical cancer, the EMBRACE II trial is now the standard of care, and evidence has demonstrated significant improvements in local control and survival with advanced radiation techniques [14]. Brachytherapy is an integral part of radical cervical cancer treatment and is used for recurrent endometrial cancers as well [3-5]. Anatomical variability, post-surgical distortion, and extensive lateral disease often preclude ideal geometry with standard applicators alone [6,7]. In such scenarios, selective hybrid techniques improve high-risk clinical target volume coverage while sparing normal tissues [9]. In addition, 3D-printed brachytherapy increases flexibility in needle placement, especially for large tumors [15,16]. Although new commercially available templates and 3D printers have revolutionized patient-specific applicator selection, challenges persist regarding equipment cost and practitioners' training, especially in low- and middle-income countries. The use of freehand needles guided by tactile and visual feedback is a feasible alternative, but there is still limited published data about the utilization of this technique [17]. Our case series depicts three challenging cases where inadequate target coverage by available templates guided us to use freehand needles (Table 1).

**Table 1:** Clinical characteristics, brachytherapy techniques, dosimetry, response, and toxicity of the three cases. FIGO, International Federation of Gynecology and Obstetrics; CCRT, concurrent chemoradiotherapy; EBRT, external beam radiotherapy; BT, brachytherapy; HR-CTV, high-risk clinical target volume; D90, dose received by 90% of the HR-CTV; D2cc, dose to the most exposed 2 cm^3^ of the organ at risk; EQD2, equivalent dose in 2-Gy fractions; SIB, simultaneous integrated boost; RTOG, Radiation Therapy Oncology Group.

Parameter	Case 1	Case 2	Case 3
Age (years)	68	45	65
Diagnosis/FIGO stage	Recurrent high-grade endometrioid adenocarcinoma of endometrium; IIIB	Recurrent moderately differentiated squamous cell carcinoma of cervix; IIIA	Poorly differentiated squamous cell carcinoma of cervix; IIIC
Disease site/extent	Deep right vaginal wall recurrence involving middle two-thirds of vagina	Vaginal stump recurrence extending to lower third of vagina, labia and clitoris	Large cervical mass involving middle third of vagina with extensive left parametrial involvement to pelvic sidewall
Prior treatment	Hysterectomy; 4 cycles carboplatin/paclitaxel followed by CCRT (45 Gy/25 fractions + weekly cisplatin)	Subtotal hysterectomy followed by CCRT (45 Gy/25 fractions + weekly cisplatin)	Pelvic EBRT 45 Gy/25 fractions with SIB to 55 Gy for enlarged pelvic lymph nodes
Brachytherapy technique	Freehand interstitial technique with 3 needles placed directly into right vaginal wall lesion and stabilized with Styrofoam	Hybrid technique using vaginal cylinder with interstitial needles for vaginal disease and 3 direct freehand needles targeting clitoral disease; Styrofoam stabilization	Hybrid technique using Fletcher–Suit applicator with left-sided freehand needles targeting deep parametrial disease; Styrofoam stabilization
Dose/fractionation	7 Gy × 4	7 Gy × 4	7 Gy × 4
HR-CTV D90 (EQD2)	94.2 Gy	88.2 Gy	81.2 Gy
OAR D2cc (EQD2)	Bladder: 66 Gy; Rectum: 60.9 Gy	Bladder: 83 Gy; Rectum: 74 Gy; Sigmoid: 73.5 Gy	Bladder: 82 Gy; Rectum: 75 Gy; Sigmoid: 74.5 Gy
Clinical/radiological response	Complete response on MRI at 3 months; mild vaginal wall fibrosis	Substantial reduction in vaginal and clitoral disease at completion of BT and 3-month follow-up; MRI response assessment unavailable	Partial response at 3 months with 2.5-cm residual cervical mass; complete response on MRI at 6 months
Follow-up/disease status	Local disease-free until October 2025; subsequently developed pulmonary metastases and received palliative systemic therapy	Last documented follow-up November 2025	Alive and disease-free at latest documented follow-up in December 2025
Toxicity	Vaginitis treated conservatively; mild vaginal wall fibrosis	Purulent vaginal discharge at last follow-up, treated with antibiotics	Grade 2 proctitis (RTOG), managed with dietary modification, steroid enemas, and argon plasma coagulation

For Case 1, the Kelowna perineal template or a 3D-printed applicator would have been more suitable, but due to unavailability, free needles were used under tactile guidance and resulted in adequate target volume coverage and complete response. Case 2 presented as advanced tumor growth with rare vulvar involvement. We could not find any such case reported in the literature, so after detailed discussion among radiation oncologists and medical physicists, we decided to use free needles to cover visible vulvar disease. A good clinical response was noted. However, we could not document a radiological response, and incomplete follow-up is a limitation for reliable radiological response and clinical toxicity.

Case 3 was unsuitable for standard tandem and ovoid template due to extensive lateralized tumor extension. We were able to get adequate target coverage by combining a Fletcher-Suit applicator and multiple free needles. The dosimetric constraints were difficult to meet, and rectum and sigmoid received EQD2 approaching 75 Gy, although our planning aim was to achieve 65 Gy for each. The patient later presented with grade 2 proctitis requiring interventions. We learned that focusing on hard-organ-at-risk constraints is safer compared to soft constraints.

Our experience suggests that hybrid interstitial brachytherapy combined with freehand needles can provide sufficient dosimetric coverage and improved local control in carefully selected patients with large, lateralized tumors, extensive parametrial and unusual vulval involvement; however, its use requires caution. The patient should be educated about expected benefits and potential risks of the innovative procedure along with treatment limitations before obtaining informed consent. As there are risks involved, the procedure should be carried out at high-volume centers with experienced teams. Evaluation of long-term outcomes and late toxicity is needed to further clarify this approach.

We acknowledge limitations in our data, including small sample size, retrospective nature, and limited long-term follow-up.

## Conclusions

The case series shows that freehand interstitial needle placement was technically feasible in three complex gynecologic cancer cases where conventional applicators or available templates were inadequate. Personalized hybrid brachytherapy using freehand interstitial needle placement represents an adaptable, cost-effective alternative in settings where dedicated applicators are unavailable and usual templates are inadequate in carefully selected patients treated by experienced teams with image-guided planning and strict attention to OAR constraints. With meticulous planning and adherence to dosimetric principles, this approach may provide acceptable target coverage and favorable clinical outcomes. Larger studies with standardized dosimetric reporting, longer follow-up, toxicity assessment, and comparison with standard hybrid applicators or 3D-printed templates are needed before wider adoption.
